# Dealing with the COVID-19 pandemic: an opportunity to reflect on sustainability research: May 29 2022

**DOI:** 10.1007/s00550-022-00528-w

**Published:** 2022-08-02

**Authors:** Benjamin Nölting, Bettina König, Anne B. Zimmermann, Antonietta Di Giulio, Martina Schäfer, Flurina Schneider

**Affiliations:** 1 Research Center [Sustainability—Transformation—Transfer], Eberswalde University for Sustainable Development, Eberswalde, Germany; 2 Integrative Research Institute on Transformations of Human-Environment Systems (IRI THESys), Humboldt Universität zu Berlin, Berlin, Germany; 3 Centre for Development and Environment, University of Bern, Bern, Switzerland; 4 Program Man-Society-Environment (MGU), University of Basel, Basel, Switzerland; 5 Center for Technology and Society (ZTG), Technische Universität Berlin, Berlin, Germany; 6 Institute for Social-Ecological Research (ISOE), and Faculty of Biological Sciences, Goethe University of Frankfurt, Frankfurt, Germany

**Keywords:** COVID-19, Corona pandemic, Sustainable development, Sustainability research, Interdisciplinarity, Transformation, Reflection

## Abstract

The COVID-19 pandemic has jolted societies out of normality, possibly creating new conditions for sustainability transformations. What does this mean for sustainability research? Because of the scope of the crisis, researchers have been heavily involved: not only have they had to speed up the pace of scientific production to provide urgently needed COVID-19 knowledge, but they have also been affected citizens. For sustainability science, this calls for an experience-based reflection on the positionality and orientation of research aiming to support sustainability transformations. Twenty sustainability researchers discussed their sustainability research on COVID-19 in three workshops based on the following questions: How does the pandemic—and the measures taken to deal with it—affect sustainable development? What can we learn from the pandemic from the perspective of societal transformation? The present discussion paper emerged from this multidisciplinary exchange among sustainability researchers, considering five topics: impacts of the COVID-19 crisis on sustainability transformations; learning for sustainability transformations; the role of solidarity; governance and political steering; and the role of science in society. Our discussions led to a meta-level reflection on what sustainability research can learn from research on COVID-19 regarding topics and disciplinary angles, time dimensions, the role of researchers, and how adequate preparation for both crises and long-term transformations requires interdisciplinary interaction.

## Introduction

Despite the existence of pandemic plans in all EU member states and early Sars-COV‑2 warnings (ECDC 2007; Lopreite et al. 2021; Maxmen 2021), the COVID-19 pandemic has been perceived and interpreted by the broad public in Europe as a very disruptive incident. Consequences are affecting all fields of activity and are given high media visibility. They affect all social groups and sectors worldwide in a way never experienced in younger history by many, including (sustainability) scientists (e.g., Deutscher Ethikrat 2020). The pandemic and the measures that have been taken to respond to it have upset daily lives, exacerbated vulnerabilities, and greatly challenged what we had taken for granted individually and societally—at least temporarily. The pandemic is a complex and global problem that has repeatedly been requiring immediate solutions, although many unknowns and uncertainties about cause-and-effect relations make action often problematic. It has led to a major joint effort across disciplines, social groups, sectors, and nations, as well as to massive interventions that would have been unimaginable for many, especially in the Global North, just a few weeks before the onset of the crisis.

This has caught the attention of sustainability researchers who have been investigating levers for and barriers to systems change for several decades (e.g., Feola 2015; Grin et al. 2010; Westley et al. 2013). In a fast-forward mode, reactions to the pandemic display many characteristics that are similar to how problems in the context of sustainable development are dealt with. Why? Sustainability problems are *also* both local and global; they are complex, permeate all fields of society, and lead to trade-offs; and solving the problems calls for drastic and far-reaching changes. Accordingly, while sustainability researchers acknowledge the suffering that COVID-19 has caused and is still causing, they also see the pandemic and the responses to it as an opportunity to look at this “fast-forward” process with the aim of analysing a new constellation and its impact on societal transformations towards sustainability. At the same time, it has become clear that the measures taken to respond to the pandemic can have both a positive or negative impact on sustainability. On the one hand they have imposed changes to distinctly unsustainable patterns of behaviour and consumption (e.g., reduced mobility), on the other they have uncovered and challenged social dynamics and values (e.g., social inequalities and how they impact people’s exposure to risk, understandings of democracy, etc.). Two questions emerge from these observations regarding the relation between the COVID-19 pandemic and sustainability: First, what can we learn from the pandemic from the perspective of the societal transformation required by Agenda 2030 (UN 2015)? Second, how does the pandemic—and the measures taken to deal with it—impact sustainable development?

Such concerns have led sustainability researchers to the question whether the COVID-19 crisis might be a window of opportunity for fostering sustainability transformations (e.g., Schot 2020). Several contributions from (sustainability) research have already reported on first empirical knowledge, discussed how sustainability and the COVID-19 pandemic relate, and provided policy recommendations about how measures taken to deal with the current crisis should be designed in a way that supports sustainability transformations (e.g., Kleve et al. 2020; Vinke et al. 2020; https://www.wpn2030.de/corona-und-nachhaltigkeit, https://www.csc-blog.org/de, https://www.praefaktisch.de/covid-19/). Research projects have spontaneously emerged, revealing sustainability scholars’ desire to further explore the different levels and domains where the COVID-19 pandemic relates to sustainability. These projects often adopt an interdisciplinary, transdisciplinary, and transformative perspective, with the aim of generating systems, target, and transformation knowledge for social-ecological change (ProClim/CASS 1997).

As of June 2020, a group of such sustainability researchers in the German-speaking part of Europe engaged in a spontaneous explorative exchange about whether the pandemic, the responses to the pandemic, and societal reactions to it also have an impact on sustainability research. They addressed such questions as: Does the pandemic uncover processes and dynamics that research should focus on? Does it challenge established methodological approaches? Does the pandemic reveal blind spots of sustainability research or point out phenomena or processes that until now had been underestimated? Does it question our assumptions about the role of science in societal transformation?

The present discussion paper offers the result of a structured exchange on ongoing sustainability research projects and in-depth and iterative reflection about lessons to be learned from research exploring sustainability impacts of the pandemic that took place from June 2020 to April 2021. The paper presents insights from this reflection process on sustainability research and the COVID-19 pandemic at two levels: At a first level, it offers insights from sustainability researchers (Sect. 2) working on projects investigating COVID-19 phenomena from a sustainability perspective. All the researchers who contributed to this section are aware that the pandemic is still evolving and that we are far from grasping what its effects will be in the long term. Nevertheless, the assumption of this paper is that analysing how the COVID-19 pandemic is being handled will provide impulses for improving governance and management of major social-ecological crises. Taking this into account the paper aims to explore areas of tension, to clarify what topics and questions related to the COVID-19 pandemic are of particular relevance to sustainability research, and to identify what may be learned from the pandemic for sustainability and for management of sustainability problems.

At the second level—drawing on the polyphony of statements made by the researchers who contributed their reflections, experiences, and assessments at the first level—the paper’s main author team reflects upon how the pandemic might inform sustainability research (Sect. 3). We are primarily interested in the question how sustainability research needs to be oriented and adjusted to new conditions, and how it can be further developed and adapted. Indeed, it would be inappropriate to offer a synthesis of the statements offered by the individual researchers in Sect. 2, since these statements are based on work in progress and reflect the multiplicity of foci, approaches, and positions that can be found in the community of sustainability researchers. Instead, what we provide is a meta-level reflection on the fitness for purpose of sustainability research in COVID-19 times and some open questions we believe need to be tackled by the sustainability science community. Importantly, in this discussion paper we limit our considerations to the social and scientific contexts of the Global North, especially of the German-speaking part of Europe.

## A multidisciplinary exploration of sustainability researchers’ positions and concerns

The discussion paper emerged from three workshops (Table 1) carried out by a network of researchers who are committed to sustainability research and who work in Switzerland (CH), Germany (D), and Austria (A) (see Annex: Table 2). In the first workshop in October 2020, participants conducting research on the impacts of the COVID-19 crisis on sustainable development presented preliminary results (see Annex: Table 3). This generated a lively expert debate about both the suitability of research approaches to investigate the impacts of the pandemic and the validity of research results gained during the pandemic. For this reason, the main author team of the discussion paper organised a second workshop in December 2020 with the purpose of discussing such meta-level questions. Based on the minutes of the first workshop, the main author team identified key areas and questions to discuss further.

**Table 1:** Three workshops on sustainability research on COVID-19 topics ^a^

1)	“Online Mini-Conference on Corona and Sustainability. 2nd networking meeting”, 29 October 2020, organized by the Centre for Development and Environment, University of Bern, 44 participants (see list of presented projects: Annex, Table 3; an initial networking meeting had taken place on 4 June 2020 with the purpose of identifying potential common interests of sustainability researchers.)
2)	“Theses and key questions for social-ecological research on Corona”, 14 December 2020, organised by the Eberswalde University for Sustainable Development, 20 participants
3)	“Online Mini-Conference on Corona and Sustainability. 3rd networking meeting”, 27 April 2021, jointly organized by the Centre for Development and Environment, University of Bern, and the Research Center [Sustainability—Transformation—Transfer], Eberswalde University for Sustainable Development, 23 participants

^a^ The term “Corona” is commonly used in German-speaking areas for COVID-19. We have kept it where co-authors used it

In the follow-up process to the second workshop, some of the participants formulated answers to the identified key questions and thus contributed to the present paper. Contributors were explicitly invited to provide insights, observations, and concerns, regardless of whether these were backed by systematic research. Their statements had to meet three criteria: (1) They should mirror the perspective of a scientist and not be a personal statement. (2) They should answer one of the following questions: “What does the Corona pandemic show that we did not see before?”, “What are topics/questions that we could not have investigated without the pandemic (not at all or not in the same way)?”, and “With regard to what questions of sustainability research can we gain insights from the pandemic and how it is dealt with?”. (3) They should not exceed 1000 characters.

The main author team discussed the statements and clustered them. If one of the statements did not meet the criteria, the team reached out to the author(s) and asked for a revision. Beyond this, the main author team did not influence the content of the statements. Thus, the paper also includes statements with which the main author team might not agree. The statements reflect the perspective and thinking of the authors who provided them; clustering of the statements did thus not occur with the aim of integrating them in a synthesis.

After having clustered the statements, the main author team treated these statements as qualitative data and discussed commonalities, differences, and fields of tension. This discussion was informed by the following question: What are the implications of the Corona pandemic and the lessons learned from the Corona pandemic for sustainability research? In a third workshop in April 2021, the meta-level reflections and questions of the main author team were discussed with the authors of the statements and subsequently developed further.

The procedure adopted by the main author team in developing this discussion paper is thus informed by the strategy of triangulation and of discursive validation: The statements provided by the sustainability researchers and the conclusions drawn from comparing them were discussed among the main author team until agreement was reached. The contributing researchers were provided with, and had the possibility of reacting to, both the conclusions of and the text introducing each cluster of statements. The following clusters emerged from the first workshop and were discussed in the second workshop: 1) impacts of the COVID-19 crisis on sustainability transformations, 2) learning for sustainability transformations, 3) the role of solidarity, 4) governance and political steering, and 5) the role of science in society. Each of these five topics is presented in the following sections with a) a brief substantiation of its relevance, b) key questions, c) the statements formulated by the workshop participants, and d) a short interim résumé.

### Impacts of the COVID-19 crisis on sustainability transformations

In recent years, sustainability research has increasingly stressed that fundamental transformations of socio-ecological systems are needed to combat the great challenges of our time, especially climate change, biodiversity loss, and social inequalities (e.g., Köhler et al. 2019; Schneider et al. 2019). Transformations involve deliberate, emancipatory, and systemic changes in dominant worldviews, practices, institutions, power relations, ecosystems, physical infrastructure and resource flows (Feola 2015; O’Brien 2011; Westley et al. 2013). Existing research has shown that such transformations are highly complex and contested, and that they rarely unfold in linear ways. Ecological, social, and economic dimensions need to be considered, as well as their interrelations, short and long-term dynamics including path dependencies, and rebound and overcompensation effects. It has also been suggested that crises and external shocks—despite their often tragic implications—might serve as windows of opportunities to catalyse sustainability transformations (Nohrstedt et al. 2021) However, there are still rather limited insights into how transformations occur, and how decision-makers can govern these profound change processes for truly advancing sustainable development (EEA 2015).

The crisis caused by the Corona virus can be regarded as an external shock that fundamentally affects the global health, economic, social, and environmental systems to an extent and at a speed that no one had ever foreseen. To reduce the spread of the virus, governments all over the world imposed hygiene measures, a limitation of social contacts, and reduced mobility. As a consequence, schools, restaurants, stores, child care, recreational facilities, transportation facilities, etc. had to reduce their operations, close temporarily, or run virtually, and citizens as well as organizations had to reorganise themselves. This in turn influenced various subsystems that are important from a sustainability perspective, such as mobility, energy use, nutrition, industrial production, education, care, the use of public space, and political debates. Deficits of existing systems became clearer and some experiences with adapting them were garnered.

Based on these insights, the following question arises:

- *How is sustainable development challenged by the COVID-19 crisis and what windows of opportunity emerge for fundamental transformations at a system level?*

#### Contributions with possible answers and further reflections

**Roman Grüter, Institute of Natural Resource Sciences (IUNR), Zurich University of Applied Sciences, CH.** Controlling short-term health and economic impacts of the pandemic are currently the first priorities. This creates a risk that the political and research focus shifts away from the more long-term impacts of climate change and environmental degradation. At the same time, there is a unique chance to identify, assess, and scale up bottom-up initiatives that emerged during the crisis, with a potential to contribute to sustainable development (e.g., related to digitization, solidarity networks, or sufficiency lifestyles). Such initiatives evolved with a high level of creativity based on the experiences made during the pandemic. To unfold their full potential, there is a need to carefully assess their ecological, social, and economic impacts through participatory research. To build back better after COVID-19, we should not be misled by conservative and preservationist forces and attitudes expressing a wish to go back to pre-Corona times, and thus risk missing the “window of opportunity” offered by the crisis.

**Livia Fritz, École Polytechnique Fédérale de Lausanne (EPFL), Laboratory for Human Environment Relations in Urban Systems (HERUS), CH.** The COVID-19 crisis has heavily disrupted entire systems of everyday practices, for example in the field of mobility: remote work has reduced everyday mobility; lockdowns have transformed domestic spaces into places of work, care, and recreation; safety concerns have led to a decreasing use of public transport. While it is too early to draw conclusions regarding the crisis’ longer-term effects, the current rupture opens a window of opportunity for reflecting on, experimenting with, and observing new mobility practices and ways of fostering them. Numerous cities have put in place ephemeral infrastructure projects (e.g. pop-up cycling lanes, pedestrian zones) with the intention of favouring low-carbon mobility such as cycling and walking while enabling spatial distancing. These infrastructural interventions and the contestations and resistances they are provoking provide sustainability scientists with a unique opportunity to explore in real time how city planning measures can leverage sustainability transitions in rapidly changing environments.

**Bettina König, Eberswalde University for Sustainable Development, D.** The COVID-19 pandemic has also challenged the economic system as a whole and generated fundamental uncertainties. Businesses of different sizes are struggling to survive the crisis despite governmental relief packages. However, some sectors have also won, maybe even in the long term, such as regional, organic food and bicycle businesses. Hence, some businesses have succeeded in translating the fragility created by the crisis into a window of opportunity for a stronger sustainability orientation, whereas others have not. To understand under what conditions the currently unsustainable economic system can be transformed towards sustainability, in a first step empirical research must analyse how national and regional restrictions and financial support measures, (temporal) changes in consumer practices, ongoing transformation processes, entrepreneurial creativity and decision-making, digitalization, and sustainability and other strategies explain these economic developments. These insights might strengthen societies’ capabilities for sustainable development after Corona.

**Anja Bierwirth, Wuppertal Institute, Climate, Environment and Energy, D**. The pandemic crisis has not only challenged the overall economic system, it has also revealed aspects of social disparities such as inequalities in living standards and housing quality, educational prospects or generally low salaries in essential professions caring for society’s basic needs. Overall, these topics are not new, but they have appeared with a new urgency in light of the pandemic. As such, intensified discussions on these topics in the media, social media, and in politics can be seen as a window of opportunity enhancing the development of new sustainable development strategies: strategies that embrace equality aspects of social and health systems, improvements in public health and healthy living environments including infrastructure for more active mobility (e.g., pop-up bike lanes), and fairer distribution and higher quality of public space.

**Rico Defila & Antonietta Di Giulio, Program Man-Society-Environment (MGU), CH.** The pandemic uncovers issues of relevance to sustainability (e.g., social inequalities, negative effects of globalized provisioning systems, negative impacts of accelerated life-styles based on high and long-distance mobility). But these insights will not support sustainability transformations unless we dig deeper: The measures to fight the spread of the virus are meant to be temporary. They are imposed. They target behavior. Their purpose is to fight an urgent problem with the explicit promise that the status quo as it was will come back. That is, they do not address deep leverage points and they do not provide an opportunity for reflecting about a desirable future. The probability that measures will lead to actual change seems smaller than that everything will switch “back to normal”. To transform the pandemic into a window of opportunity, discussions about deep leverage points allowing for reflection and change of the dominant frame should be initiated and supported (e.g., the limited ability of the current economic system to solve problems, the societal role and responsibility of the government).

*Short interim résumé*: The contributions point to far-reaching effects of the pandemic in several fields of socio-economic systems, e.g., mobility, economy, and urban development; in many cases they reveal problems already known, not only to sustainability research. Government regulation, changes in (consumer) behaviour, economic crises, new initiatives, government subsidies, etc. have shaken up constellations and redistributed risks and opportunities, affecting citizens, households, and companies in very different ways, increasing profits and power for some. But for others with few resources, social inequality and injustice are worsening. It is not possible at this stage to assess the impact of these changes on sustainable development. What should be closely monitored is whether and how the changes are deep and enduring ones, and how they can be shaped. So far, short-term crisis management and its effects seem to predominate; work on other, longer-term crises seems to be pushed into the background.

### Learning for sustainability transformations from adapting practices and acquiring competences in dealing with the COVID-19 crisis

Sustainability transformations require changes not only on a systemic level, but also deep and comprehensive changes of practices on an individual and organizational level covering all areas of daily life such as work, food, mobility, housing, and recreation. Research on how practices are established and on the challenges of changing daily routines has shown that practices result from the interplay of practical know-how, social negotiations, social norms, and value systems, as well as material arrangements and provision systems (Brand 2010; Di Giulio et al. 2014; Reckwitz 2002). Single practices such as going to work by car are closely interlinked with other practices such as bringing children to school, doing the daily shopping, or accessing recreational facilities. Changing a single practice has complex effects, and single practices are therefore difficult to change. Since “knowing” is not enough, informational measures aimed at convincing people of the necessity to change daily routines in order to achieve sustainability have little success. Research also shows that life events such as the birth of a child or relocating bear the chance of changing routines in reaction to new individual and social demands (Schäfer and Jaeger-Erben 2012). Due to the restrictions caused by the COVID-19 crisis most people had to adapt their private and working routines drastically in a very short time, without being able to “prepare” for these changes. Depending on their private and work situation individuals experienced these changes rather differently. For sustainability researchers this situation offers an opportunity to address the following question:

- *Does the COVID-19 crisis offer us chances to modify daily routines in the long term, to benefit from experiences with adapted practices, and to acquire transformational competences?*

#### Contributions with possible answers and further reflections

**Franziska Stelzer & Carolin Baedeker, Wuppertal Institute, Climate, Environment and Energy, D.** At present, the population is strongly affected emotionally and feels the immediate consequences of the pandemic. It has not yet been possible to generate such concern for global climate issues, although they are just as threatening in many scenarios. One assumption is that climate effects are not (yet) as “visible”—at least in the Global North: Attribution is difficult, since we cannot really distinguish normal weather fluctuations from real climate impacts. Direct negative impacts on our lives, our friends, relatives, or family are not yet perceptible. Moreover, political actors are not acting with the same urgency and not as consistently regarding climate themes. Another hypothesis is that the prospect of a “normal” life after the pandemic makes it easier for people to accept harsh measures. Climate protection measures, on the other hand, would not be limited in time. Nevertheless, with support from climate experts, policy-makers could use a similar appeal for climate issues: the aim of the measures is to protect “every human being” on a global scale.

**Flurina Schneider, Institute for Social-Ecological Research (ISOE), and Faculty of Biological Sciences, Goethe University of Frankfurt, D.** The Corona crisis forced many people to change their practices, sometimes with positive sustainability outcomes. Should they start to enjoy these changes, the new practices might stabilize. I assume that this might become true for business trips, which might partly be replaced by virtual communication in the long term, because organizations and employees will have experienced the benefits of saving time and resources. But there is also a high risk that people will prefer to just restore the pre-Corona status and return to their original practices as soon as possible. This might be the case for recreational mobility, which is heavily missed by many. Hence, whether daily routines will be modified in the long term, depends on the way the changes correspond to individuals’ value systems and on whether the crisis will have been able to trigger value changes. Fostering debate on these values can be an important contribution of sustainability research.

**Martina Schäfer, Center for Technology and Society (ZTG), Technische Universität Berlin, D.** As already mentioned, people had to adapt and re-arrange their daily routines in many areas very suddenly, and they had to react iteratively to several changes in restrictions. For some this situation led to experimentation with new practices such as going more often by bike and on foot, taking advantage of nearby possibilities for recreation, going shopping on outdoor weekly markets, intensifying their gardening or do-it-yourself activities, all of which have positive sustainability potentials. Whether people had the chance to use this situation to reflect on habitual practices and whether they appreciated the possibility of making new experiences, depended a lot on the potentials and constraints of their professional and private circumstances. Time resources as well as competences to deal with sudden changes and the social network to be able to cope with challenges seem to have been decisive factors. Further insights into these decisive factors and supportive measures that foster stabilization of newly acquired practices, could be very helpful for research on sustainability transformations.

**Laurenzia Karrer & Lilian Julia Trechsel, Centre for Development and Environment, Education for Sustainable Development Cluster, University of Bern, CH.** The Corona crisis is widening the inequality gap also for the ability to learn and change. While for some, it triggered moments of transformative learning because (social) stress decreased and people’s focus changed from outward to inward, others found themselves confronted by new restrictions and obligations in addition to their limited resources. Education is a good example: The rapid shift to online learning, e.g., in higher education, put wealthier pupils at an advantage. We also know that transformative learning for sustainable development is only possible when learners feel empowered to self-reflect, which seems easier to foster face-to-face. Experiences made during the Corona crisis help us understand how learners can be empowered, inequalities be addressed, and new spaces for learning be created. Whether individuals find the creativity to deal with the crisis and have the space for change, depends strongly on their resources and on their options for action. Guaranteeing adequate access to digitalization is only one of several crucial requirements.

*Short interim résumé*: Discussions about preliminary results in this thematic area highlighted the danger that sustainability researchers may draw premature conclusions based on first analyses and reflections on changes in practice caused by the COVID-19 crisis. Conclusions like “individuals are willing—and able—to change their practices drastically from one day to the other” or “people have discovered the positive sides of leisure activities in their neighbourhood/going by bike/spending their holidays in their home country” cannot be drawn at this point in time. Sustainability researchers must be aware of the risk of overestimating the lastingness of temporary changes in practice; they should draw a clear line between interpretation of data and wishful thinking. However, there are indications that major social differences are influencing the possibility of perceiving the crisis situation as an opportunity for reflection, learning, and experimentation. While for some, this seems definitely to be the case, for many, the crisis is simply exacerbating existing precariousness. Thus, political measures that address issues of inequality and environmental justice as well as provide supportive social and material infrastructure are still underestimated or too undifferentiated in strategies for fostering sustainability transformations.

### The sudden prominence of solidarity and its role in sustainability transformations

Equity and justice are values enshrined in the Brundtland definition of sustainable development (UN 1987). But it took a paradigm shift in the global sustainability debate to make the common good a more fundamentally and jointly defined principle in the UN’s *Agenda 2030* (UN 2015). By contrast, sustainability research has always included justice (e.g. “environmental justice”); and the study of the commons has gained in importance (Biermann et al. 2020; BUND and Misereor 1996; Ostrom 1990, 2007). However, the value of solidarity emerged rather late on the research agenda, e.g., with concepts of solidarity economy and care economy. With the COVID-19 crisis, solidarity reached sudden prominence as a striking individual and community experience and topic (Morin and Abouessalam 2020). Immediately after the first lockdown, waves of solidarity spread, with people who had never met before joining forces to provide multiple services, sometimes revealing great creativity in making the impossible possible. In most cases, no institutional regulatory measures curbed this creativity.

The mere idea of imposing a lockdown to preserve a population’s health and health system is an institutional expression of solidarity. The key value of “accessibility of health services for everyone” was given a prime role in decision-making; in addition, governments in wealthy countries provided massive funds to buffer the impact of economic stalling. At the same time, they struggled to obtain medical equipment needed by their own countries at the expense of international solidarity; and citizens started doubting the validity of governmental intentions.

This shows the tensions between the competing values of justice and solidarity versus individual freedom and prosperity that underlie sustainable development. How can these trade-offs be addressed? Sustainability research is likely to benefit from exploring how individuals, society, and public policy act regarding the common good and solidarity, and how challenges of implementing solidarity in informal and formal institutionalized ways were taken up from the local to the global levels during the COVID-19 crisis. Against this background, the following key question was brought up:

- *How does institutionally implemented solidarity function in the COVID-19 crisis and what can we learn from this for sustainability transformations?*

#### Contributions with possible answers and further reflections

**Franziska Stelzer & Carolin Baedeker, Wuppertal Institute for Climate, Environment and Energy, D.** The COVID-19 pandemic drastically reveals the complexity of interdependencies when trying to achieve a “sustainable”, “just”, and “healthy” city. On the one hand, the challenges of demographic change became very clear (loneliness in modern societies, isolation of the elderly because of the protection measures). On the other hand, many initiatives emerged that strengthened solidarity in society and cohesion in the crisis. People started using their spare time to go shopping for at-risk patients or to hold video calls with grandparents. But the COVID-19 pandemic also affects other vulnerable groups. Lower-income households are feeling the effects of housing, energy, education, mobility, and digital poverty more severely than before. This is also evident regarding socio-ecologically spatial differences, which vary greatly: In times of the pandemic, higher-income households have retreat options in their gardens, near parks or forests, and are also less exposed to noise and air pollution. There is therefore a need for strengthening solidarity at the structural and spatial levels, e.g., by integrating environmental justice aspects systematically into the planning of more resilient post-Corona cities.

**Holger Kreft, freelancing consultant and project developer at Büro für zukunftsfähige Regionalentwicklung (Wuppertal), D.** Understanding how the mainstream economic paradigm works is perhaps the most important lever for solving unsustainable developments. Everything is assumed to be scarce, while human needs are supposedly unlimited. Everything is commodified and the world is exploited for unlimited growth. Among other things, this way of thinking and living stabilizes deficits in equity by reproducing and reinforcing their structural conditions. This creates a lack of basic security for almost everyone. COVID-19 is now challenging many routines at school and work. Inequalities are being exposed as well as exacerbated. We see more clearly who is disadvantaged by the distancing rules and who is underprivileged anyway. Solidarity actions try to counteract this and express how people wish to live together. They indicate that the system could be repurposed by the pandemic should we decide to promote this vision. The old paradigm is confronted with new and more progressive ones, but also with even older and more regressive ones.

**Andreas Kläy & Anne Zimmermann, Centre for Development and Environment, University of Bern, CH.** The Corona crisis has led to exceptional processes of decision-making among governments, both in terms of limiting freedom of movement and providing massive support for everyone. Political dissent was set aside in favour of finding solutions for everyone. This seems due to the sudden generalized insight that it was essential to uphold countries’ health systems and save lives. We seem to be facing a global commons issue: the subsistence of the virus anywhere in the world remains a menace for global human health and all countries depend on one another. Thus, theoretically, if we want to be able to deal adequately and sustainably with the pandemic, we need to ensure that solidarity works at the global level; at the same time, solidarity must also work at the local and national levels, and be in agreement with the global level. The* Global Sustainable Development Report* argues that the global *environmental* commons are one of six entry points for leveraging sustainability transitions; can we add *health* as part of these commons?

**Lena Bloemertz, University of Basel, CH.** The temporal scale is important as well when reflecting on solidarity. Corona has changed from being discussed as a sudden emergency to a lingering problem. This has made discussions around solidarity and the trade-offs between different impacts of Corona and handling them more complex. Observations of increasing discontent with measures demanding individual solidarity contrast with an increasingly professional and institutionalised solidarity (e.g., efforts for equal distribution of vaccines). While a generalised evaluation of solidarity levels would necessarily be misleading, I suggest that Corona allows us to get a glimpse of the difference in disaster response between a rapid disaster and a slow onset/creeping disaster. Sudden onset disasters have been shown to trigger a more rapid and widely supported response than slow onset disasters. Having collectively experienced this change in the Corona crisis might provide lessons for better understanding solidarity and willingness to change in the wake of creeping disasters (such as climate change).

*Short interim résumé: *To conclude, voluntary compliance with strict restrictions to prevent the spreading of COVID-19 is essential from an epidemiological viewpoint, but difficult for citizens to maintain. The strong experience of solidarity and the awareness of latent inequalities fostered acceptance of restrictions in the first wave of the epidemic. But acceptance of restrictions conflicts with democratic principles and expectations of individual freedom. Thus, implementing institutional forms of solidarity to achieve equal access to resources (e.g. health) as a fundamental principle of sustainable development requires more than a focus on individual behaviour: it requires a value-based change in societal and economic structures. COVID-19 sheds light on the role of solidarity in coping with a crisis and on how solidarity can or could be institutionalized as a value in local, national, and international governance, as a means of moving towards sustainable development. It will be interesting to explore further how people’s consent regarding the key importance of solidarity can be fostered.

### Governance and political steering

Social-ecological research addressing the role of the state and public policy for sustainability transformations predominantly emphasizes long-term governance processes (Grin et al. 2010, WBGU 2011). In contrast, the COVID-19 pandemic sheds light on short-term political crisis management. This broadens the potential future analytical view of sustainability scholars on public policy from crisis management and adaptation policies to precautionary policy and prevention, in line e.g. with climate policy (Glass and Newig 2019; Köhler et al. 2019; Turnheim et al. 2020). The COVID-19 pandemic allows for observing whether and how public policy links crisis management with adaptation policy and reflexive governance. Three aspects are interesting here (Nölting et al. 2019).

First, the articulation and aggregation of interests and ideas is crucial for sustainability governance (power dimension). The COVID-19 pandemic sheds light on *mechanisms of power.* What interests prevail in the distribution of subsidies? Who benefits from and who is affected how by COVID-19 measures? Second, the *state’s ability to function and act* determines how policy programs for sustainable development are designed and implemented (capacity dimension). During the ongoing COVID-19 crisis some states are mobilizing enormous resources—and at the same time are confronted with the limits of state control, at least in the context of liberal democratic states. Third, the design of the *policy process* influences the orientation of policy towards sustainability. Reflexive governance, which includes dealing with uncertainty, policy learning, legitimacy, and acceptance, is conducive to this (process dimension). The Corona pandemic shows that most citizens accept even far-reaching measures, provided these measures are explained transparently and the goals are seen as meaningful. On the other hand, they are critical of the fact that participation of citizens and parliaments has been heavily restricted. Therefore, it is interesting to discuss how crisis management could be complemented by elements of reflexive governance and participation. A key question is:

- *What can be learned from the political management of the COVID-19 pandemic for governing sustainability-oriented transformations?*

#### Contributions with possible answers and further reflections

**Basil Bornemann, Sustainability Research Group, University of Basel, CH.** When the severe consequences of the pandemic became apparent, a debate unfolded on whether the crisis should be managed only through restorative crisis management, or whether the state should orient its crisis management towards sustainability. Some even argued that the crisis offered an opportunity to initiate a longer-term transformation policy. The Corona crisis is thus a litmus test for the sustainable state, as it shows whether and to what extent governments respond to sustainability considerations under increased stress conditions and take them into account in their actions. To what extent and under what conditions have governments succeeded in integrating sustainability aspects into COVID-19 crisis management? How do governments pursue a sustainability-oriented crisis management that incorporates short- and long-term considerations? These questions are all the more relevant as sustainability-oriented governance faces an ongoing social-ecological crisis in the wake of the Anthropocene.

**Benjamin Nölting, Eberswalde University for Sustainable Development, D.** During the ongoing Corona crisis, public policy in different countries is setting priorities in very different ways. On the one hand, a “strong state” with a strong executive is focusing on rapid crisis management to prevent Corona from spreading—and sometimes tends to be autocratic. On the other hand, a laissez-faire policy has left crisis management to the market and civil society—without support for vulnerable groups. It is interesting to observe what objectives public policy has aimed at while managing the Corona crisis and how it has considered implications for dealing with long-term social-ecological crises. The German Advisory Council on Global Change recommends a proactive state that sets sustainability priorities, while at the same time offering its citizens extended opportunities for participation and opening options for the economy for sustainable activity. How does a proactive state link crisis management with precautionary sustainability policy? Is the EU’s Green Deal an appropriate example of proactive policy?

**Anja Bierwirth, Wuppertal Institute for Climate, Environment and Energy, D.** It is astounding how rapidly policies and regulations have been implemented due to the pandemic. This is all the more remarkable as these measures seriously affect persons, societies, and economies in a hitherto mostly unexperienced and often severe way. Nevertheless, regulations have been accepted by a broad majority in society. The high intensity of public intervention stands in apparent contrast to non-decisiveness in other areas with a need for action, such as climate change. Many political actions in this area are not taken due to a presumed rejection by society or economies, although their impacts would not be nearly as severe as the impacts of the restrictions imposed during the Corona crisis. The hazards due to a changing climate are—just like the pandemic—a serious threat broadly acknowledged in society. So if the perception of a hazardous situation obviously supports the acceptance of necessary action, might this also be true for climate action? With the experience of the Corona crisis, implementing more courageous climate policies seems to be not only necessary but also possible.

**Basil Bornemann, Sustainability Research Group, University of Basel, CH.** Corona has shown that in pluralistic, liberal societies, massive restrictions of individual liberties are possible and acceptable, at least if accompanied by financial compensation. However, it also shows that such interventions reach limits of legitimacy and effectiveness in the medium and long term, for example, when necessary social, mental, and financial resources are exhausted. In this respect, Corona crisis management shows that governance by limitation and compensation alone are not sufficient strategies for a much more long-term-oriented sustainable state. The shaping of sustainability-oriented transformations calls for complementary governance approaches such as the strengthening of social empowerment and deliberation. While social empowerment encourages people to act and strengthens their sense of efficacy and autonomy, deliberation improves the transparency of governance and creates a robust basis for dealing with the uncertainty involved in social change. Perhaps these approaches to deepening democracy could enable liberal democracies to deal with the pandemic in a more effective manner.

**Bettina König, Eberswalde University for Sustainable Development, D.** Due to their nature, pandemics have a spatial dimension and all governance levels are needed to shape options for deciding, implementing, and controlling measures. How decision makers organize learning in times of crisis across governance levels from local to global, what barriers they encounter and what new governance solutions occur, is of relevance to sustainability issues as well. With climate change, crisis situations are to be expected, like floods or heat waves. Governance research on the Corona pandemic, starting from pre-existing pandemic management plans to capturing crisis experiences, could thus inform how crisis plans related to climate change and sustainability could be improved and how governance learning can be organized in times of crisis. This could involve (1) the role of competition between nations or regions on governance learning and (2) learnings about behavioral Corona management measures (e.g. physical distancing, hygiene) compared to natural science and “technological fix” approaches (e.g. vaccination).

**Holger Kreft, freelancing consultant and project developer, Büro für zukunftsfähige Regionalentwicklung (Wuppertal), D.** Communication between politicians and citizens on public issues is a key aspect of governance; they communicate mainly indirectly via different channels. During the pandemic, indirect communication has played an even greater role. Therefore, the question arises: What mindsets do politicians reveal in their communication, and what mindsets do citizens show? For various reasons, few politicians communicate publicly about what their own conception of humanity and society is, what exactly their values are—i.e. to what extent they can handle feedback, understand their own emotions, accept scientific evidence, respect diversity, tolerate ambiguity, and really take responsibility. What story do they believe in and are they able to tell to support positive change? As a result, citizens can hardly understand what considerations guide politicians’ proposals. Different mentalities among politicians and citizens clash, without any clarity emerging about the causes of these differences. In principle, however, a further development of mindsets is possible if political communication bears this in mind.

*Short interim résumé: *In the COVID-19 crisis numerous states are demonstrating their ability to act by imposing strict regulations, providing health services, and distributing financial compensation. At the same time, the limitations of public policy and state resources are becoming obvious. Public policies for managing the health crisis pursue specific goals, are linked with other policy fields, and engage citizens in various ways. Financial redistribution, reference to long-term sustainability goals, and the design of the policy process including participation, learning, communication, and deliberation influence future pathways for sustainable development. The above contributions suggest the need for improving our understanding of how to integrate short-term crisis management into long-term governance for sustainability transformations and at the same time improve the adoption of social empowerment and reflexive governance for crisis management.

### The role of science in society

Sustainability research is largely driven by an interest in gaining comprehensive insights into the human-environment relationship and by the desire to provide knowledge that is beneficial for transformations towards sustainability. Sustainability scholars want to have a voice and societal impact. Engaging in collaborations with policy-makers and other stakeholders, doing research in “real-world” settings by designing and implementing interventions, and integrating the knowledge and expertise of non-academics in participative approaches, are crucial features of sustainability research. Accordingly, what role science should have in policy-making, how to manage the science-policy interface, and how to define non-academic expertise have been important topics in the scholarly debate (Bäckstrand 2003; Cash et al. 2003; Defila and Di Giulio 2019; Hastie 2007; Schneidewind and Singer-Brodowski 2014).

Against this background, many occurrences in the wake of the “COVID-19 crisis” have caught the eye of sustainability scholars. First, research has received unprecedented public attention. In some national contexts, findings have fed directly into political decision-making, in others they have been ignored. Researchers have achieved celebrity status or been threatened because of their findings. Second, there was an immediate demand for communicating scientific findings but the exposure of the academic mechanisms of validating (and revising) findings has caused profound confusion. Researchers have been blamed for not providing clear guidance or for trespassing by trying to impose measures. They have fought against being urged to provide conclusions or have been offended by not being listened to. Third, public attention has focused on some disciplines, resulting in a reductionist problem framing of the complex “COVID-19 crisis”. Last but not least, a diversity of self-proclaimed experts with questionable expertise have entered the stage, raising the question about how to distinguish valid from invalid non-academic knowledge. Against this background, the question of interest for sustainability research is:

- *What can (transformative) sustainability research learn from the role played by academia during the pandemic and from the general processes that took place in the research-policy interface?*

#### Contributions with possible answers and further reflections

**Livia Fritz, École Polytechnique Fédérale de Lausanne (EPFL), Laboratory for Human Environment Relations in Urban Systems (HERUS), CH.** Characterised by great urgency, uncertainty and complexity, the COVID-19 crisis has put the spotlight on science and its role in policy-making. These experiences with science-policy interactions cast new light on scientific *uncertainty and dissent* and what they mean for political decision-making and narratives of control. The COVID-19 crisis shows that when it comes to complex problems scientists point to unknowns and rarely speak with one voice, thus necessitating an interrogation of the hierarchy of expertise in deciding which voices are (not) heard, and a questioning of political agility in rapidly evolving situations. This offers learning potential for how to acknowledge the provisional and contested nature of knowledge without giving rise to public scepticism and political inaction. A debate on how scientific knowledge, normative judgments, and democratic decision-making (should) interact can provide invaluable insights for collectively tackling future crises, most notably the climate crisis.

**Flurina Schneider, Institute for Social-Ecological Research (ISOE), and Faculty of Biological Sciences, Goethe University of Frankfurt, D.** Sustainability scientists should chime in and raise their voices about how to manage the pandemic (e.g., to ensure that economic recovery packages consider sustainability values). But there is more to learn: When the pandemic started, the Swiss federal council mandated a task force of scientists helping to provide up-to-date scientific insights needed to overcome the crisis. But the ad-hoc collaboration between scientists and authorities has not been easy. Scientists have repeatedly publicly urged the authorities to listen to science, whereas the authorities have defended the political decision-making process and accused the scientists of too narrow a perspective. Hence, it has become obvious that science-policy collaborations must be soundly established *before* an acute crisis and that there is a need to mutually agree on modalities. Learning from this experience for sustainability calls for the establishment of a permanent task force consisting of sustainability scientists, the authorities, and further key stakeholders.

**Antonietta Di Giulio & Rico Defila, Program Man-Society-Environment (MGU), CH.** Many issues at the research-policy interface have indeed been exposed in the response to the Corona pandemic and are of interest to sustainability research as well. This includes methodological issues in need of being addressed given the extent of conspiracy theories, self-proclaimed expertise, and willingly produced “fake news” that are circulating: If we want to stick to transdisciplinary approaches in sustainability research and include non-academic expertise and knowledge, what are appropriate answers to the erosion of the notions of knowledge and expertise? In addition, there is a need to reflect on specific knowledge and validity-related issues that refer to how the sustainability research community has been reacting to the Corona crisis: How justified are conclusions (in books and papers) about the state of society after the Corona crisis, drawn while the crisis is still evolving (or worse: in the first months of the crisis)? How do we avoid conclusions about the sustainability impact of measures in response to the pandemic from being biased by either wishful thinking and/or our own system of values?

**Bettina König, Eberswalde University for Sustainable Development, D.** Besides these fundamental questions, the “sustainability scholar perspective” in the Corona pandemic offers an additional learning. For understanding and managing the pandemic across all societal systems, different and sometimes fractioned forms of academic knowledge were and are needed. It would be interesting to reconstruct how different disciplinary scientific perspectives played a role in different phases. From an outside perspective, the first phase was dominated by the natural sciences (and “not so prestigious disciplines” such as epidemiology and applications of “not so new” models as of the early 1970ies made relevant contributions). Later, economic expertise was required—and only later were education, care, sociology, psychology, ethics, law, and culture publicly declared as missing in the discussion. From our field’s perspective this raises the question how interdisciplinarity can be established within academia as more than “a luxury”. How can interdisciplinarity become generally acceptable as the form of science that enables more holistic reaction to urgent knowledge gaps in times of crisis? This involves communication about uncertainties, not-knowing, and ambiguity in a way that supports societal coping capabilities in times of crisis.

*Short interim résumé: *One topic the above contributions deal with is the question of what can be learned from the science-policy interaction that has been established during the COVID-19 pandemic, with a view to developing a more effective science-policy interface for sustainability. The contributions also indicate what questions surfaced in the public debate about the pandemic regarding understandings of knowledge, integrating a scientific and a political rationale, and ascertaining a comprehensive approach to complex problems—and how these questions can also be eye-openers in the context of sustainability. Finally, and on a tone that is more self-reflective, the contributions raise the question whether sustainability research could learn something from how the community of sustainability scholars reacted to the pandemic.

## Meta-level reflection: What can sustainability research learn from research on COVID-19?

We used critical debate, confronted diverse research perspectives and concerns triggered by COVID-19, and reflected on our short- and long-term experiences, examining sustainability research (projects) from different individual positions and perspectives as presented in Sect. 2. The aim of the multidisciplinary exchange among the German-speaking sustainability researchers involved in this paper was to gain orientation in a new societal dynamic that challenges sustainability—and sustainability research.

Based on our expertise on the complexities involved in human-environment interactions, we assume that the COVID19 pandemic has made certain aspects more visible, but it may also have (indirectly) triggered impacts that will only emerge and manifest themselves in the following endemic period. A synthesis aiming to offer new research avenues would not be appropriate in the midst of the prolonged crisis and its anticipated endemic phase, and across such a broad, disparate, and very incomplete spectrum of topics (see Annex: Table 3). For this reason, we do not want to jump to premature conclusions based on these insights, nor postulate a research program for sustainability scholars on COVID-19.

Nevertheless, the discussions reported above showed that the pandemic has challenged our scientific habits and questioned implicit assumptions we had about sustainability research. Based on concrete research experiences, the polyphonic reactions and thoughts (Annex: Table 2) led to a shared reflection on the meta-level about the nature and role of sustainability research, not only during but also, presumably, after the pandemic. The main author team’s first overarching ideas were therefore discussed with contributors at the third workshop (see Table 1). Here, we organise this reflection around three generic issues under which the discussion threads can be subsumed: a) the thematic and disciplinary focus of sustainability research, b) its time dimension, and c) the personal involvement of sustainability researchers. Each issue ends with questions that might be helpful for contextualising, (re-)orienting, and further developing the topics, methods, and value orientation of sustainability research in the longer term.

- *Thematic and disciplinary focus: Which topics and disciplines are relevant in acute crises such as COVID-19, for scientific analysis and evidence-based dealing with the respective crisis?*

The statements in Sect. 2 show a need for sustainability research to deal with—or more systematically acknowledge—research topics that gained visibility during the pandemic. As a health (and not an environmental) crisis, the COVID-19 pandemic has shed light on the fact that global crises—and policies to deal with them—bear the danger of increasing social inequalities. We observed that the need to react very quickly led to risk prevention measures that did not adequately take into account impacts on the most vulnerable social groups. Besides well-known social inequalities (e.g. gender, differences in income and education), others, so far not as visible in the sustainability debate, emerged as requiring urgent attention to be able to cope with the crisis (e.g. quality of housing and the living environment, increased infection risk due to type of employment). The issue of increased social inequalities becomes even more striking when looking at the global situation. Apart from the well-known unequal-access-to-health-resources issue in the Global South, new concerns have emerged, e.g. reactions to the pandemic have destabilized informal economies that buffered rampant poverty, hunger, and other livelihood insecurities of the most vulnerable populations (Carmody et al. 2020; Kesar et al. 2021; Khambule 2020).

As the other side of this thematic coin, solidarity emerged as an important topic that can be dealt with on different levels by sustainability research. Reduction of individual freedom to protect the most vulnerable groups was widely accepted in the first wave of the pandemic as a form of institutional implementation of solidarity in Europe. Additionally, different individual and collective forms of solidarity arose spontaneously to support those societal groups that were most affected by the pandemic. They also emerged to alleviate social injustice or provide ad-hoc help. A systematic reflection on formal and informal institutionalizations of solidarity may deliver insights into new modes of governance, also in the context of sustainability science.

Altogether, the topics of social inequalities and solidarity show that there are no simple solutions to wicked problems, and sustainability research must be careful not to focus too early on specific issues: It must remain alert to side-effects and problem shifts. How can we make sure that we consider social issues when choosing a focus triggered by a health crisis? And are social aspects such as the accessibility and resilience of basic public services, as well as their inclusiveness and preventive character, appropriately considered? Moreover, these examples lead to the question whether sustainability research adequately reflects on *how* social science perspectives are related to other research perspectives, in particular in the natural sciences. Are they considered sufficiently in problem framing and analysis, as well as in the development of strategies and solutions to deal with these problems? Is the relationship between the different disciplines taken into account while working on interdisciplinary research questions?

b) *Time dimension: How can sustainability research deal with both urgent crises and keeping a long-term transformation perspective?*

Sustainability research aims to support *transformation*, which is radical and long-term in nature, rather than *changes in the short-term* that can be changed back again. The COVID-19 crisis has shown that acute crises call for feasible short-term solutions even if they generate trade-offs. Massive state intervention and redistribution of income and chances, among other measures, will set the course for the future. This experience reinforces the challenge for sustainability research of developing concepts that are effective in the short term but also viable in the long term, thus sticking to the basic direction of a long-term transformation perspective. In light of the COVID-19 pandemic, new questions arise for sustainability research with regard to state policy and the design of political processes under crisis conditions: Do we have time for participation, learning, solidarity, dealing with uncertainty, and (reflexive) science? How can crisis management be coherently linked with long-term sustainability goals?

Further, the statements in Sect. 2 illustrate sustainability researchers’ assumptions about changes due to the COVID-19 pandemic. It will be important to monitor very closely what changes are only temporary and which ones have a long-term and potentially definitive (transformative) character. Before generalizing, sustainability scientists must be careful not to overestimate the importance of certain phenomena that might be of a temporary nature or only valid for rather small parts of the population.

After all, the statements in Sect. 2 demonstrate the need for examining empirical evidence to understand how the overall societal constellation for sustainability transformations has changed. Contributions and discussions during the third workshop revealed a need for updated systems knowledge: Did the leverage points for sustainability transformations change due to COVID-19? Did the pandemic uncover hidden leverage points? Did it create new ones? Researchers must reflect on whether they are still focusing on the relevant short-term phenomena and are asking the right long-term questions to co-develop robust solutions for sustainability transformations. In particular, when drawing conclusions about their results, researchers need to be sensitive to the time horizon and to how short and long-term research perspectives relate to each other.

c) *Personal involvement: What can sustainability researchers learn from the challenge of being both affected by COVID-19 and keeping an analytical distance to it?*

The question of (sustainability) scholars’ personal involvement in the phenomena they study, e.g. climate change, has long been the focus of debate (Gilford et al. 2019; Head and Harada 2017). Concerns have been raised that being personally affected leads to loss of critical distance for scientific analysis. Therefore, explaining one’s position regarding normative aspects and dealing consciously with emotions triggered by global threats are integral parts of sustainability research. When sustainability researchers rely on scientific methods to achieve analytic clarity of understanding, they ideally aim to make their position transparent and contextualise it (Schneider et al. 2019).

The fact that scholars are both affected by the pandemic and part of the ongoing changes that they are investigating can in fact be an opportunity for sustainability scholars to hone the skills needed for interdisciplinarity, in particular those skills leading to the epistemological awareness needed to conduct their work purposefully and with an open mind for other scientific perspectives. As underlined in the statements about learning processes in Sect. 2, the need to cope with changes in professional and private life causes emotions and may foster self-reflection. From the perspective of scientific practice, emotions and self-reflection may seem to interfere with “dispassionate” and “objective” analysis; but they can also be seen as part of the tacit knowledge through which we can connect with the value dimension of sustainability research and with the societal challenges of transformation. However, this requires clarifying what good scientific practise can acknowledge and making sense of emotional aspects that gain importance in times of crisis, e.g. influences from opinions, emotions, interests, power, and knowledge gaps.

Questions emerged from our multidisciplinary reflection on how to further develop our scholarly tacit knowledge out of the lived changes and challenges: Are we repeating the same things as before the COVID-19 crisis, although the preconditions have changed? Are we only able to see what we are trained to see? Science and society need critical, reflexive mechanisms for correcting perceptions and mindsets (Nölting and König 2019). Beyond the COVID-19 pandemic, times of crisis may lead sustainability scholars to (re-)focus their self-conception of engaged scholarship, thus benefitting from a more systematically reflected research dimension that could be called a “lived sense of knowing” based on wisdom. This might allow for another quality of field access and insights that may foster a (re-)balanced production and communication of “robust” knowledge and help to reveal wishful thinking and blind spots easily overlooked in sustainability research. But these ambitions also lead to the following questions: What structural conditions, personal capabilities, and mechanisms do we need to engage both in preventive and crisis coping research to achieve appropriate social interaction, individual, and societal learning? And what best supports knowledge generation, communication, and transfer in this complex endeavor? Exchange among researchers of the kind we presented here may be an appropriate way for critically reflecting on the effects of being affected while doing sustainability research.

To round off this discussion, let us come back to the starting question: What can sustainability research learn from the study of the COVID-19 pandemic? We began our multidisciplinary exchange after half a year of living with the pandemic and submitted our contribution for the first time after one year. Now, looking at the pandemic after over two years we share the impression that a deep and broad reflection on the many meanings and impacts of the pandemic for society, economy, and the environment is needed.

Empirical and other studies on the impacts of the pandemic have been published meanwhile (e.g. Echegaray et al. 2021; OECD 2020). Our own discussion with scholars and organisations from other scientific fields can be connected with finished, ongoing, or planned studies, e.g. in Germany (Deutscher Ethikrat 2022; Leopoldina 2020, 2021; Walz et al. 2022), in Switzerland (Rühli and Thier 2021; Beyeler et al. 2021; SNSF 2021), and in Austria (ÖAW 2020a, b), even if the main perspective of these studies is not sustainability science. Indeed, their publications reveal similar reflections and questions, e.g. what is needed for acute crisis management, and reference to the concepts of resilience, vulnerable groups, inequalities, and complex multiple crises. Sustainability science has focused on these topics for some time already and can contribute knowledge for preventing and handling future crisis in societies (Walz et al. 2022; wpn2030 2020). Vice versa, sustainability scientists can learn from pandemic-related debates in other domains about the role of science in society, science communication, research(ers) being affected (emotionally) by crisis, the limits of science, the role of and conditions for inter- and transdisciplinary research, etc. (Deutscher Ethikrat 2022; Leopoldina 2021). It remains to be seen what questions will be taken up from these studies; we hope that our multidisciplinary reflection can contribute to the overall debate on lessons to be learned from the pandemic.

A final word seems necessary in light of the current military crisis in Europe and its global impacts: our call for being better prepared for crises as sustainability researchers and our suggestions are more important than ever. This should include an awareness that lingering crises (e.g. poverty, climate change, loss of biodiversity) are also in need of an anticipatory and preventive attitude (wpn2030 2020). In times of acute crisis, inter- and transdisciplinary sustainability research is not a luxury and just one perspective among others; rather, it is a fundamental need that relies on critical and thorough reflection on short-term measures and developments with the lens of the needed long-term transformation.

## Appendix

**Table 2:** Overview of the disciplinary academic training of the authors and contributors (alphabetical order)

Name	Disciplinary academic training (university diploma and PhD)
Carolin Baedeker	Diploma: geography PhD: geography, social sciences
Anja Bierwirth	Diploma: architecture; Master of Science: environmental sciences
Lena Bloemertz	Diploma: geoecologist PhD: social geography
Basil Bornemann	Diploma: environmental sciences PhD: political science Venia legendi: political science & sustainability research
Rico Defila	Diploma: law
Antonietta Di Giulio	Diploma: philosophy, German studies PhD: philosophy
Livia Fritz	Diploma: development studies, political science PhD: sustainability sciences
Roman Grüter	Diploma: environmental sciences PhD: environmental sciences
Laurenzia Karrer	Diploma: social and communication sciences, sociology
Andreas Kläy	Diploma: forest engineering
Bettina König	Diploma: horticultural sciences PhD: agricultural sciences
Holger Kreft	Diploma: geography PhD: waste management
Benjamin Nölting	Diploma: history, political science, economics PhD: political science
Martina Schäfer	Diploma: biology PhD: environmental technology, sociology
Flurina Schneider	Diploma: geography, botany, environmental protection, law PhD: sustainability sciences, geography
Franziska Stelzer	Diploma: psychology, economics PhD: economics
Lilian Julia Trechsel	Diploma: education for sustainable development PhD: geography and sustainable development
Anne B. Zimmermann	Diploma: English and American literature, French literature, German literature PhD: English languages and literatures, postcolonial and cultural studies

**Table 3:** Overview of projects presented at the 29 October 2020 online mini-conference

Project title	Researchers and affiliations; other details provided
SUSBECT: SUStainable BEhavior during and after Corona Times	Dominik Georgi, Marcel Zbinden, Larissa Dahinden, Carmen Grebmer Institut für Kommunikation und Marketing (IKM), HSLU, Switzerland April 2020–May 2022 Report in German
Nachwuchsgruppe PuR: Präsentation “Ernährungsgewohnheiten und Verpackungsaufkommen im Kontext der Corona-Pandemie”	Elisabeth Süßbauer, Klara Wenzel, Anne Müller Center for Technology and Society, TU Berlin, Germany May 2019–April 2024; COVID-19 was included in the project as of March 2020 http://www.pur-precycling.de
Corona als Chance oder Gefahr für Bildung für Nachhaltige Entwicklung?	Laurenzia Karrer, Lilian Trechsel, Anne Zimmermann Centre for Development and Environment, Univ. of Bern, Switzerland May-Dec 2020 Paper prepared for the COVID-19 Round Table at the Higher Education Summit 2020, 1 Sep 2020: “What has the Coronavirus crisis done to teaching?“
Konzeption und Umsetzung einer Solidaritätsplattform—Partizipation und Wissenschaft im Quartier Arrenberg, Wuppertal	Franziska Stelzer Wuppertal Institut, Climate, Environment and Energy, Germany May 2020–June 2021 SolPlat: Plattform für Quartierssolidarität: gemeinwohlorientierte Ansätze zum Aufbau pandemie-resilienter Quartiere nutzen
Corona-Krise und soziale Innovationen in Unternehmen und Organisationen	Patrick Baur, Stephanie Moser, Michèle Egger, Alina Ferrara Centre for Development and Environment, Univ. of Bern, Switzerland May-Dec 2020 (unpublished report)
Logbuch der Veränderung	Bettina König, Benjamin Nölting Research Center [Sustainability—Transformation—Transfer], Eberswalde University for Sustainable Development March 2020—ongoing https://logbuch-der-veraenderungen.org/
ReZeitKon: Zeitverwendung, Zeitwohlstand und Konsum während der Corona-Maßnahmen	Sonja Geiger, Ulf Schrader, Stefanie Gerold TU Berlin, Germany September 2018–August 2021; the project leaders conducted an additional survey during the pandemic. http://www.rezeitkon.de/wordpress/de/ergebnisse/
Aus der Corona-Krise für die Klimakrise lernen: Voraussetzungen für das Übertragen von Wirksamkeitsüberzeugungen aus dem Lock-Down auf die Bewältigung der Klimakrise	Stephanie Moser; Sebastian Seebauer Centre for Development and Environment, Univ. of Bern, Switzerland; Joanneum Research, Graz, Austria May 2020—ongoing First results presented at ICEP 2021 (5–8 Oct, Siracusa)
